# Broad and durable protection against SARS-CoV-2 and SARS-CoV by an intranasal chimpanzee adenovirus vaccine expressing tandem RBDs and nucleocapsid

**DOI:** 10.1371/journal.ppat.1014436

**Published:** 2026-07-24

**Authors:** Yaping Liu, Runhong Zhou, Tian Zhao, Ruoke Wang, Kun Zhu, Junxian Hong, Ziqing Yang, Qianqian Yang, Yuqing Lei, Yang Bai, Jing Wei, Peng Chen, Xinyu Fan, Qi Zhang, Xuanling Shi, Peng Liu, Zhiwei Chen, Linqi Zhang

**Affiliations:** 1 Comprehensive AIDS Research Center, Pandemic Research Alliance Unit, Center for Infection Biology, School of Basic Medical Sciences, Tsinghua University, Beijing, China; 2 AIDS Institute, Department of Microbiology and Pandemic Research Alliance Unit, School of Clinical Medicine, Li Ka Shing Faculty of Medicine, The University of Hong Kong, Pokfulam, Hong Kong Special Administrative Region, People’s Republic of China; 3 Centre for Virology, Vaccinology and Therapeutics, Hong Kong Science and Technology Park, Hong Kong Special Administrative Region, People’s Republic of China; 4 Department of Clinical Microbiology and Infection Control, The University of Hong Kong-Shenzhen Hospital, Shenzhen, People’s Republic of China; 5 School of Biomedical Engineering, Tsinghua University, Beijing, China; 6 Department of Rheumatology and Clinical Immunology, Peking Union Medical College Hospital, Chinese Academy of Medical Sciences & Peking Union Medical College, Beijing, China; 7 School of Basic Medical Sciences, Tsinghua University, Beijing, China; 8 Institute of Biopharmaceutical and Health Engineering, Tsinghua Shenzhen International Graduate School, Tsinghua University, Shenzhen, China; 9 Institute of Biomedical Health Technology and Engineering, Shenzhen Bay Laboratory, Shenzhen, China; Erasmus Medical Center, NETHERLANDS, KINGDOM OF THE

## Abstract

Current intramuscular COVID-19 vaccines reduce severe disease but offer limited protection against infection, enabling viral persistence, immune escape, and transmission. We developed intranasal rare-serotype chimpanzee adenovirus 68 (AdC68)–based vaccines expressing heterologous tandem receptor-binding domains from SARS-CoV-2, SARS-CoV, and MERS-CoV, fused to the SARS-CoV-2 nucleocapsid (AdC68-4RBD(XBB.1.5)-N) to enhance both B and T cell responses. Antigen integrity was confirmed by receptor binding and recognition by multiple conformation-sensitive monoclonal antibodies. In mice, intranasal AdC68-4RBD(XBB.1.5)-N elicited potent and durable mucosal and systemic immunity, including serum/saliva IgA and broad neutralizing antibodies persisting up to 40 weeks, alongside tissue-localized B and T cells. Monoclonal antibodies from long-lived bone marrow antibody-secreting cells demonstrated broad and strain-specific neutralization, providing mechanistic insight into the breadth and longevity of the antibody response. In Syrian hamsters, intranasal immunization protected against SARS-CoV-2 XBB.1.5 replication in nasal turbinates and lungs and blocked transmission for up to four months post-vaccination. Robust protection against SARS-CoV challenge was demonstrated in K18-hACE2 transgenic mice, further confirming its broad efficacy. These findings support AdC68-4RBD(XBB.1.5)-N as a promising mucosal vaccine candidate to prevent infection and transmission of pathogenic coronaviruses.

## Author summary

As SARS-CoV-2 evades immunity conferred by existing intramuscular COVID-19 vaccines and continues to evolve, there is an urgent need for novel vaccines that can effectively block viral infection and transmission. To address this, we developed an intranasal vaccine based on a rare-serotype chimpanzee adenovirus 68 (AdC68), designated AdC68‑4RBD(XBB.1.5)-N, which incorporates heterologous receptor-binding domains from highly pathogenic coronaviruses including SARS-CoV-2, SARS-CoV, and MERS-CoV, along with the nucleocapsid protein of SARS-CoV-2. In mice, intranasal immunization with AdC68‑4RBD(XBB.1.5)-N elicited broad and durable immune responses. To elucidate the breadth and durability of the antibody response, we analyzed monoclonal antibodies from long-lived bone marrow antibody-secreting cells, which demonstrated broad and strain-specific neutralization with different mechanisms. Critically, intranasal vaccination with AdC68‑4RBD(XBB.1.5)-N effectively protected hamsters from SARS-CoV-2 XBB.1.5 infection and transmission, and fully protected K18-hACE2 transgenic mice against SARS-CoV challenge, demonstrating broad protective efficacy. These findings position AdC68-4RBD(XBB.1.5)-N as a promising next-generation mucosal vaccine candidate for pandemic preparedness against pathogenic coronaviruses.

## Introduction

Current intramuscular COVID-19 vaccines face two major limitations. While these vaccines are highly effective at preventing severe disease, they provide limited protection against respiratory infection. In addition, they require frequent updates to match emerging variants, as escape mutations enable viral replication despite vaccine-induced or therapeutic antibody immunity. Both challenges stem from insufficient mucosal immunity, which permits ongoing viral replication, immune escape, and transmission. Because pathogenic coronaviruses such as SARS-CoV-2, SARS-CoV, and MERS-CoV enter primarily through the respiratory tract, vaccines that induce broad and durable mucosal immunity are essential for preventing infection and spread.

Recent efforts have explored respiratory delivery of diverse vaccine platforms, including viral vectors [1–5], recombinant proteins and nanoparticles [6–9], and mRNA–LNP [10]. While many elicited potent and protective respiratory tract immunity against SARS-CoV-2, most were evaluated only shortly after vaccination, leaving the durability of protection unclear.

Here, we aimed to develop an intranasal vaccine capable of inducing durable and protective immunity against three major, genetically distinct pathogenic coronaviruses: SARS-CoV-2, SARS-CoV, and MERS-CoV. Building on our prior work with rare-serotype chimpanzee adenovirus 68 (AdC68)–based vaccines [11–13], we engineered a recombinant AdC68 encoding heterologous tandem receptor-binding domains (RBDs) from these viruses, fused to the SARS-CoV-2 nucleocapsid (N) protein to enhance T cell responses. The RBDs were selected as the immunogens because they are the principal targets of neutralizing antibodies in infected and vaccinated humans [14–19]. Neutralizing antibodies elicited by this design are expected to block viral interactions with ACE2 (SARS-CoV and SARS-CoV-2) or DPP4 (MERS-CoV), and tissue-localized B and T cells jointly contribute to frontline defense at respiratory surfaces.

We demonstrate that the heterologous tandem RBDs preserved structural integrity through receptor-binding assays and recognition by conformation-sensitive monoclonal antibodies. In mice, comparative immunogenicity studies identified AdC68-4RBD(XBB.1.5)-N as the most potent candidate, inducing robust mucosal and systemic humoral immunity lasting up to 40 weeks, along with tissue-localized B and T cell responses. Bone marrow antibody-secreting cells (ASCs) collected 22 weeks post-prime produced broad and strain-specific neutralizing antibodies, providing mechanistic insight into the breadth and longevity of the antibody response. In Syrian hamsters, the same regimen protected against respiratory infection and transmission of authentic SARS-CoV-2 up to four months post-vaccination. Broad protection against SARS-CoV was also demonstrated in K18-hACE2 transgenic mice. These results highlight AdC68‑4RBD(XBB.1.5)-N as a highly promising mucosal vaccine candidate that could transform pandemic preparedness by providing transmission-blocking immunity and protection against diverse pathogenic coronaviruses.

## Results

### Heterologous tandem-RBDs demonstrated superior immunogenicity compared to monomeric, trimeric, or mixed RBDs

**Fig 1:**
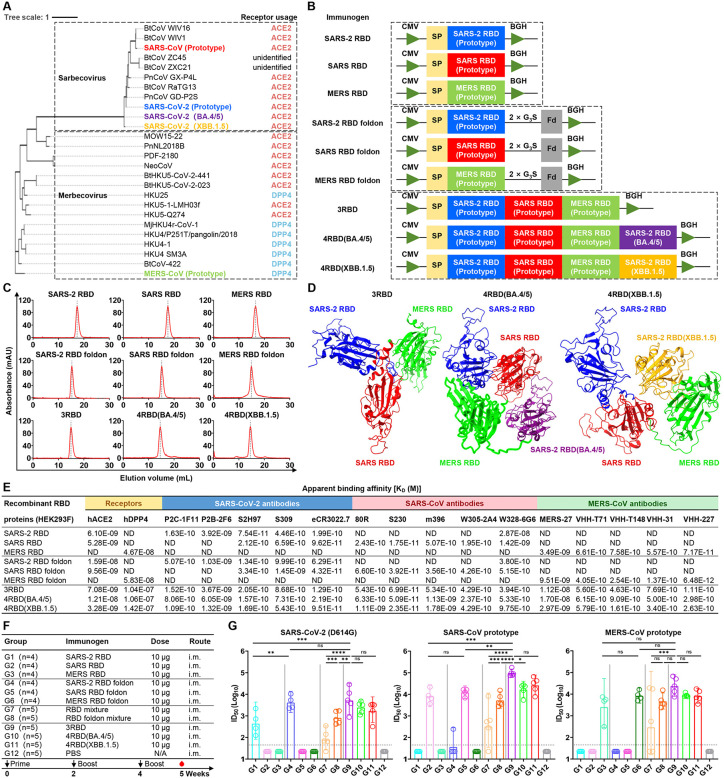
Heterologous tandem-RBDs display superior immunogenicity compared to monomeric, trimeric, or mixed RBDs. **(A)** Selection of representative RBDs from SARS-CoV-2 (prototype, BA.4/5, XBB.1.5), SARS-CoV prototype, and MERS-CoV prototype to capture the diversity of major pathogenic β-coronaviruses. **(B)** Schematic of heterologous tandem-RBDs (3RBD, 4RBD) and individual monomeric or trimerized (foldon-tagged) RBDs. Tandem RBDs were linked head-to-tail using GGGS (G_3_S) linkers and included: SARS-CoV-2 prototype (blue), SARS-CoV (red), MERS-CoV (green), BA.4/5 (purple), and XBB.1.5 (orange). SP, signal peptide; Fd, foldon tag. **(C)** Size-exclusion chromatography profiles of purified recombinant RBDs on a Superdex 200 Increase column, monitored by UV absorbance at 280 nm. **(D)** Predicted 3RBD and 4RBD structures generated by AlphaFold3 and visualized in Chimera. **(E)** SPR analysis of recombinant RBDs’ binding to human receptors (hACE2, hDPP4) and conformation-sensitive neutralizing monoclonal antibodies. ND, not detectable. **(F)** Immunization strategy for evaluating immunogenicity in BALB/c mice. The RBD mixture was a heterologous mixture of monomeric RBDs from SARS-CoV-2, SARS-CoV, and MERS-CoV, whereas the RBD foldon mixture consisted of trimeric RBDs from the same viruses. **(G)** Serum neutralizing titers (ID₅₀) at week 5 post-prime against SARS-CoV-2 (D614G), SARS-CoV, and MERS-CoV pseudoviruses. Data are geometric means ± 95% CI; analyzed by one-way ANOVA with Sidak’s test (ns, p > 0.05; *p ≤ 0.05; **p ≤ 0.01; ***p ≤ 0.001; ****p ≤ 0.0001).

Among the seven known human coronaviruses, SARS-CoV, MERS-CoV, and SARS-CoV-2 are the most pathogenic, having caused regional epidemics or global pandemics (Fig 1A). To explore whether combined or individual RBDs offer enhanced immunogenicity and protective potential, we designed three heterologous tandem-RBD constructs, designated 3RBD, 4RBD(BA.4/5), and 4RBD(XBB.1.5), by linking RBDs from SARS-CoV-2 prototype, SARS-CoV, MERS-CoV, and either Omicron BA.4/5 or XBB.1.5 in a head-to-tail fashion (Fig 1B). For comparison, we also generated monomeric and trimeric (foldon-stabilized) RBDs from SARS-CoV-2, SARS-CoV, and MERS-CoV.

All constructs were expressed in HEK293F cells, purified, and confirmed to have the expected size-exclusion profiles (Fig 1C). Structural predictions using AlphaFold3 suggested that the heterologous tandem-RBD constructs were conformationally stable and free from steric clashes (Fig 1D). Surface plasmon resonance (SPR) confirmed that both 3RBD and 4RBD retained binding specificity and affinity to human ACE2, DPP4, and multiple RBD-specific conformation-sensitive monoclonal antibodies (mAbs) (Figs 1E and S1). All tested mAbs specific to SARS-CoV-2, SARS-CoV, and MERS-CoV, including those pan-sarbecovirus-neutralizing mAbs (e.g., S2H97, S309, eCR3022.7, and W328-6G6) bound effectively to both the individual and the heterologous tandem-RBD proteins, indicating preserved epitope integrity.

To evaluate immunogenicity, 54 female BALB/c mice were divided into 12 groups (n = 4 or 5 per group) and immunized intramuscularly with monomeric (G1–G3), trimeric (G4–G6), heterologous mixtures of monomeric (G7) and trimeric (G8) RBDs from SARS-CoV-2, SARS-CoV, and MERS-CoV, tandem (G9–G11) RBDs, or PBS control (G12) at weeks 0, 2, and 4 (Fig 1F). At week 5, sera were tested for neutralizing activity against SARS-CoV-2, SARS-CoV, and MERS-CoV. While monomeric (G1–G3) and trimeric (G4–G6) RBDs induced strong autologous responses, mixed monomeric (G7) and trimeric (G8) RBDs improved breadth at the cost of potency, likely due to reduced antigen dose per RBD. Strikingly, mice immunized with 3RBD (G9) and 4RBD (G10–G11) constructs generated the broadest and most potent cross-neutralizing responses (Fig 1G). These findings highlight the preserved structural integrity and enhanced immunogenicity of tandem-RBDs, supporting their potential for broad coronavirus vaccine development.

### Generation and characterization of AdC68-based tandem-RBD vaccines for intranasal delivery

**Fig 2:**
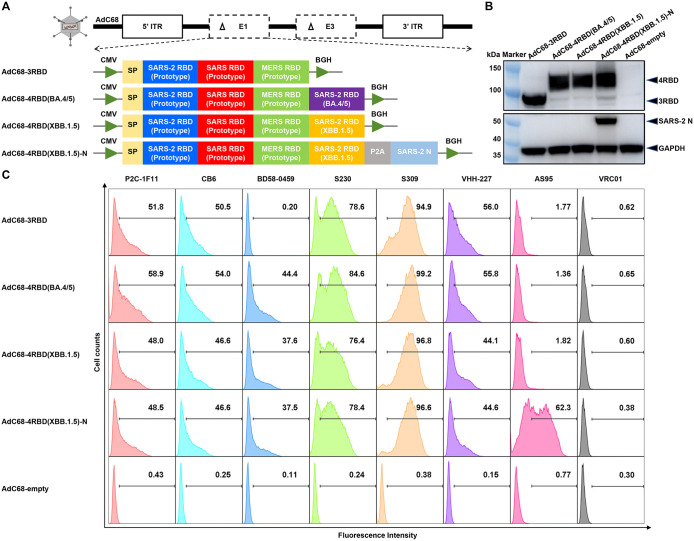
Construction and validation of AdC68 vectors expressing heterologous tandem-RBDs. **(A)** Schematic of recombinant AdC68 vectors encoding 3RBD or 4RBD (AdC68-3RBD or AdC68-4RBD), with or without the SARS-CoV-2 nucleocapsid (N) protein. Each transgene includes an N-terminal secretory signal peptide (SP) and a head-to-tail fusion of heterologous RBDs, and is driven by a CMV promoter with BGH polyadenylation. Inserts were cloned into the E1 region of the AdC68 vector via homologous recombination. Created in BioRender. Xin, S. (2026) https://BioRender.com/zy8pvgf. **(B)** Western blot showing expression of RBD and N proteins in HEK293T cells infected with AdC68-3RBD or AdC68-4RBD (10^10^ vp). **(C)** Flow cytometry confirming RBD and N protein expression using epitope-defined monoclonal antibodies: SARS-CoV-2 RBD (P2C-1F11, CB6, BD58-0459), SARS-CoV RBD (S230), SARS-CoV/SARS-CoV-2 RBD (S309), MERS-CoV RBD (VHH-227), and SARS-CoV-2 N (AS95). Empty vector and irrelevant monoclonal antibody (VRC01) served as negative controls.

To address the limited protection of current SARS-CoV-2 vaccines against upper respiratory tract infection, we aimed to induce robust mucosal and systemic immunity through intranasal immunization. To this end, we developed intranasal vaccines using the chimpanzee adenovirus vector AdC68, known for low seroprevalence and prior clinical safety [20–23]. Four recombinant vectors were generated to express 3RBD (AdC68-3RBD) or 4RBD (AdC68-4RBD) constructs, including a version of 4RBD(XBB.1.5) fused to SARS-CoV-2 nucleocapsid (N) protein (AdC68-4RBD(XBB.1.5)-N) to enhance T cell responses (Fig 2A). Transgenes were inserted into the E1 region under a CMV promoter and terminated by a BGH polyadenylation signal. Recombinant viruses were purified by CsCl gradient ultracentrifugation and viral particle (vp) titers were determined by absorbance at 260 nm. Western blotting confirmed expression of 3RBD, 4RBD, and N proteins at expected molecular weights in HEK293T cells infected with the recombinant vectors, but not with the empty AdC68 vector (Fig 2B). Flow cytometry with conformation-sensitive mAbs verified correct epitope presentation for SARS-CoV-2, SARS-CoV, MERS-CoV RBDs, and N protein (Fig 2C). These results validated the structural fidelity of the recombinant AdC68 vaccines, supporting their further evaluation in vivo.

### Intranasal AdC68-4RBD(XBB.1.5)-N induces robust and durable IgG and IgA responses

**Fig 3:**
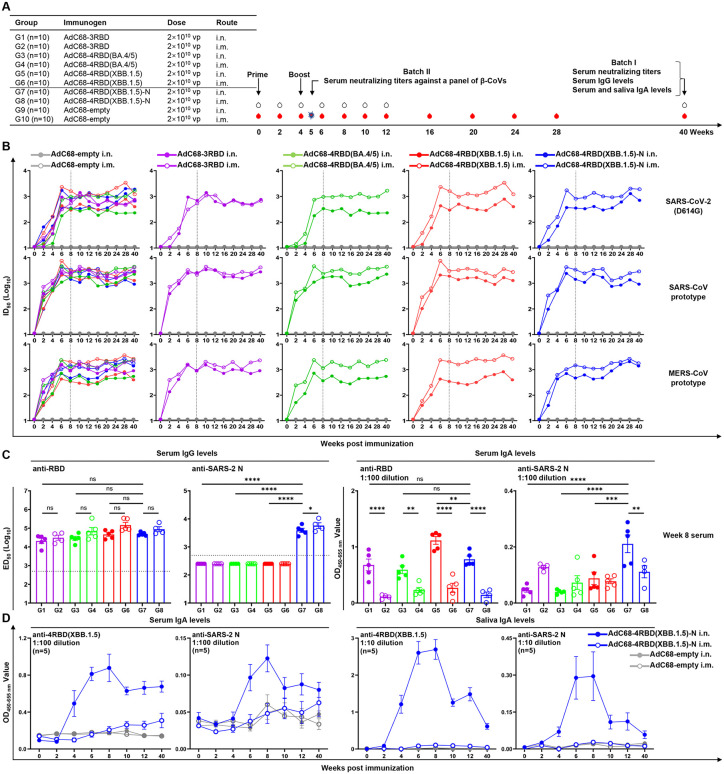
Intranasal AdC68-4RBD(XBB.1.5)-N immunization induces robust and durable systemic and mucosal antibody responses. **(A)** Immunization and sampling schedule in BALB/c mice (n = 10/group). Batch I (n = 5) was followed to week 40 post-prime for serum pseudovirus neutralization, serum and saliva binding detection. Batch II (n = 5) was analyzed at week 5 post-prime for expanded neutralization. **(B)** Longitudinal serum neutralizing titers following intranasal or intramuscular immunization against SARS-CoV-2 (D614G), SARS-CoV, and MERS-CoV pseudoviruses. **(C)** Serum binding antibody levels (IgG/IgA) at week 8 post-prime. Binding was assessed against 3RBD/4RBD and SARS-CoV-2 N proteins. Data are means ± SEM; analyzed by one-way ANOVA with Sidak’s test (ns, p > 0.05; *p ≤ 0.05; **p ≤ 0.01; ***p ≤ 0.001; ****p ≤ 0.0001). **(D)** Durable IgA responses in serum and saliva following AdC68-4RBD(XBB.1.5)-N immunization.

To compare the immunogenicity of different vaccine constructs and delivery routes, BALB/c mice (6–8 weeks old; n = 10/group) received the prime-boost immunization regimen intranasally or intramuscularly at a 4-week interval with 2 × 10^10^ vp of AdC68-3RBD, AdC68-4RBD(BA.4/5), AdC68-4RBD(XBB.1.5), AdC68-4RBD(XBB.1.5)-N, or AdC68-empty. Longitudinal sera were collected up to 40 weeks post-prime (Fig 3A). All AdC68 constructs elicited robust and durable neutralizing antibodies against SARS-CoV-2, SARS-CoV, and MERS-CoV. Titers were comparable between routes for AdC68-3RBD, whereas AdC68-4RBD constructs induced higher titers via intramuscular delivery (Fig 3B). Given the likely higher per-RBD antigen dose in AdC68-3RBD, these results suggest greater dose sensitivity of intranasal immunization, possibly due to differences in mucosal versus systemic antigen uptake and immune activation [24–26]. This aligns with the inherently higher immune tolerance of the nasal mucosa, requiring AdC68-4RBD to overcome baseline thresholds and trigger effective mucosal responses [27–30].

Binding antibody responses (IgG) to RBDs were similar across groups, while anti-N responses were observed only in AdC68-4RBD(XBB.1.5)-N–immunized mice (G7, G8). In terms of serum IgA levels, the four vaccine constructs elicited relatively comparable responses only when administered intranasally, whereas intramuscular delivery yielded minimal IgA (Fig 3C). Notably, sustained IgA responses in serum and saliva were detected in the intranasal AdC68-4RBD(XBB.1.5)-N group, persisting through week 40 (Fig 3D). These results highlight the enhanced systemic and mucosal immunogenicity of AdC68-4RBD(XBB.1.5)-N via the intranasal route.

### Broad neutralizing and tissue-localized immune responses induced by AdC68-4RBD(XBB.1.5)-N

**Fig 4:**
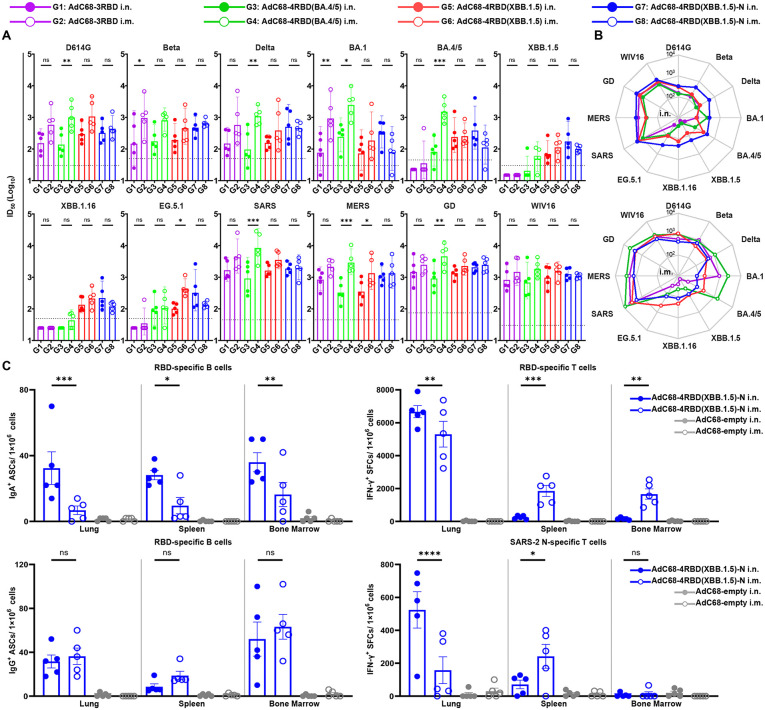
Intranasal AdC68-4RBD(XBB.1.5)-N immunization elicits broad neutralizing antibodies and tissue-localized B and T cell responses. **(A)** Serum neutralization titers against a panel of 12 pseudoviruses at week 5 post-prime. Data are geometric means ± 95% CI; analyzed by one-way ANOVA with Sidak’s test (ns, p > 0.05; *p ≤ 0.05; **p ≤ 0.01; ***p ≤ 0.001). **(B)** Radar plots of neutralizing titers (ID₅₀) following intranasal (top) or intramuscular (bottom) vaccination. **(C)** ELISPOT analysis of RBD-specific IgA^+^ and IgG^+^ ASCs (left) and RBD/N-specific IFN-γ^+^ SFCs (right) in lungs, spleen, and bone marrow. Data are means ± SEM; analyzed by one-way ANOVA with Sidak’s test (ns, p > 0.05; *p ≤ 0.05; **p ≤ 0.01; ***p ≤ 0.001; ****p ≤ 0.0001).

To assess neutralization breadth, week 5 sera from immunized mice were tested against a panel of 12 pseudoviruses. Both intranasal and intramuscular AdC68-4RBD vaccinations conferred broad neutralization, with generally higher titers observed following intramuscular delivery (Figs 4A, 4B, and S2). AdC68-3RBD neutralized 8 of 12 viruses, including bat WIV16 and pangolin GD strains, but lacked activity against recent Omicron subvariants (BA.4/5, XBB.1.5, XBB.1.16, and EG.5.1). In contrast, AdC68-4RBD constructs maintained neutralizing activity against Omicron variants, with strain-matched constructs achieving the highest titers for corresponding subvariants. For instance, AdC68-4RBD(BA.4/5) showed enhanced neutralization of BA.1 and BA.4/5, while AdC68-4RBD(XBB.1.5) and AdC68-4RBD(XBB.1.5)-N retained potent activity against XBB.1.5, XBB.1.16, and EG.5.1.

To evaluate tissue-localized responses, additional mice were analyzed at week 8. Intranasal AdC68-4RBD(XBB.1.5)-N immunization induced significantly higher frequencies of RBD-specific IgA-secreting cells in the lung, spleen, and bone marrow samples, along with elevated systemic and mucosal IgA levels. IgG ASCs were more prominent in the intramuscular group, though differences were not statistically significant (Fig 4C). Furthermore, IFN-γ–producing T cells specific to RBD and N antigens were enriched in the lungs of intranasally immunized mice (Fig 4C). These findings demonstrate that intranasal AdC68-4RBD(XBB.1.5)-N induces both broad neutralizing antibody responses and robust local B and T cell immunity.

### Intranasal AdC68-4RBD(XBB.1.5)-N induces broadly neutralizing and strain-specific monoclonal antibodies from long-lived bone marrow ASCs

To investigate the basis of the broad and durable antibody responses elicited by intranasal AdC68-4RBD(XBB.1.5)-N, we employed Modular Superhydrophobic Microwell Array Chip (MoSMAR-chip) technology to isolate RBD-specific IgG and IgA antibody-secreting cells (ASCs) from the bone marrow, a major reservoir of long-lived plasma cells. Unlike conventional B cell isolation methods, MoSMAR-chip enables antigen-specific ASC capture at the single-cell level, independent of surface BCR expression [31].

**Fig 5:**
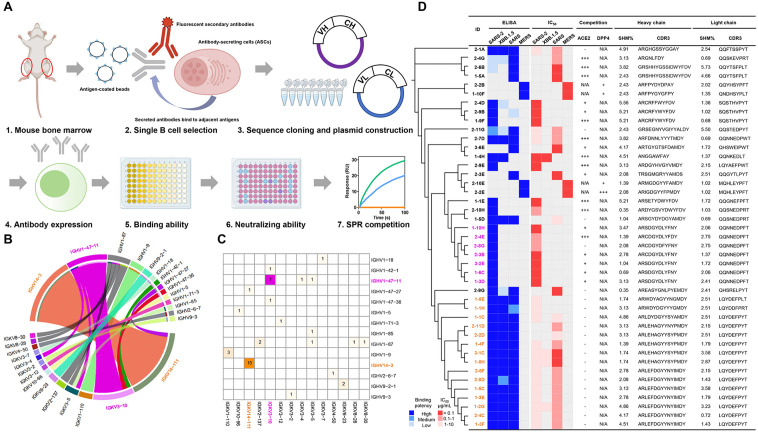
Monoclonal antibodies from bone marrow ASCs display broad and strain-specific neutralization. **(A)** Workflow for isolation and characterization of mAbs from bone marrow ASCs of an AdC68-4RBD(XBB.1.5)-N–immunized mouse. Created in BioRender. Xin, S. (2026) https://BioRender.com/7iqoere. **(B)** Chord diagram showing heavy-light chain gene pairings; arc width indicates frequency. **(C)** Heatmap of heavy-light chain usage, highlighting dominant IGHV14-3/IGKV14-111 (orange) and IGHV1-47/IGKV3-10 (pink) combinations. **(D)** Phylogenetic tree of heavy chain variable regions. Tips annotated with mAb names, binding/neutralization properties, ACE2/DPP4 competition (SPR), SHM levels, and CDR3 sequences. Competition levels: “+++” (>50%), “ + ” (10–50%), “ − ” (<10%), “N/A” (not applicable).

Bone marrow plasma cells were collected from a representative intranasally immunized mouse at 22 weeks post-prime. Following a 1-hour incubation with RBD-coated particles, RBD-specific ASCs were identified using fluorescence-labeled secondary antibodies (Figs 5A and S3A), isolated via microneedle, and subjected to VH/VL gene cloning and recombinant expression as full-length human IgG1 in HEK293F cells. Binding and neutralization properties of the resulting monoclonal antibodies (mAbs) were then evaluated.

Among the 43 isolated mAbs, 15 shared a dominant IGHV14–3/IGKV14–111 pairing (orange, Fig 5B and 5C), previously associated with sarbecovirus cross-reactivity in mosaic RBD-immunized mice [32]. Another recurrent pairing, IGHV1–47/IGKV3–10 (pink, Fig 5B and 5C), was found in 7 mAbs, with the remaining antibodies showing diverse V gene usage. Phylogenetic analysis revealed the IGHV14–3/IGKV14–111 group formed the largest cluster (Fig 5D). While all cluster members exhibited strong cross-binding, several (e.g., 3-1C, 1-8H, 1-2G) demonstrated broad neutralization against SARS-CoV-2 D614G/XBB.1.5, and SARS-CoV without ACE2 competition, and shared similar CDR3 lengths and sequences. Additional broadly binding mAbs outside this cluster (e.g., 2-1A, 2-7D, 1-5D) showed variable neutralization; notably, 2-7D competed with ACE2, suggesting an alternative neutralization mechanism (Figs 5D and S3B).

In contrast, IGHV1–47/IGKV3–10 mAbs were SARS-CoV-2–specific in both binding and neutralization, with varying ACE2 competition. Members of this group (e.g., 1-10H, 2-4E, 2-3B, 3-2E) showed ACE2 competition and strict SARS-CoV-2 specificity. A subset of mAbs exhibited strain-restricted reactivity, including SARS-CoV-specific (e.g., 1-5A, 3-6E, 2-3E) and MERS-CoV–specific mAbs (e.g., 2-2B, 1-10F, 2-10E, 2-2E). For the MERS-CoV–specific mAbs, neutralizing activity against MERS-CoV variants was retained, suggesting potential protection against the tested MERS-CoV variants (S3C Fig). Notably, mAb 1-4H potently neutralized both SARS-CoV-2 D614G and XBB.1.5 while strongly competing with ACE2, suggesting targeting of conserved residues near the receptor-binding motif (Figs 5D and S3B).

These findings demonstrate that AdC68-4RBD(XBB.1.5)-N induces a diverse and durable antibody repertoire targeting conserved epitopes, including broadly neutralizing, non-ACE2-competitive antibodies as well as strain-specific, ACE2-blocking antibodies derived from long-lived bone marrow ASCs.

To explore the neutralization mechanism of non-ACE2-competitive mAbs, we performed S1 shedding assays. These assays measure the ability of antibodies to trigger premature release of the S1 subunit from the spike trimer, a process that can render the virus non-infectious [33,34]. Our results revealed that non-ACE2-competitive mAbs, particularly those from the IGHV14–3/IGKV14–111 lineage, were able to promote S1 shedding to varying degrees (S3D Fig). Importantly, the extent of S1 shedding appeared to be associated with neutralization potency, indicating that antibody-induced spike destabilization represents a functionally relevant neutralization mechanism. These findings demonstrate that AdC68-4RBD(XBB.1.5)-N elicits a diverse and durable antibody repertoire employing multiple neutralization strategies, including ACE2-blocking and spike-destabilizing mechanisms, derived from long-lived bone marrow ASCs.

### Intranasal AdC68-4RBD(XBB.1.5)-N confers durable protection against SARS-CoV-2 XBB.1.5 infection and transmission in hamsters

**Fig 6:**
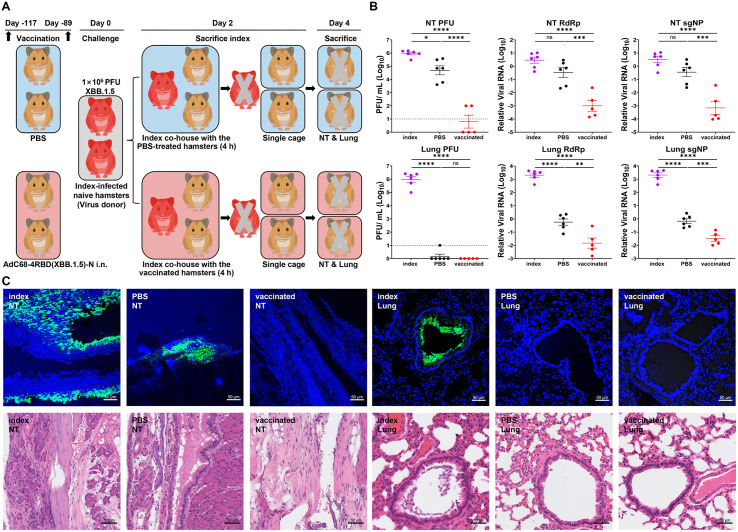
Intranasal AdC68-4RBD(XBB.1.5)-N protects against XBB.1.5 infection and transmission in hamsters. **(A)** Experimental timeline of vaccination, SARS-CoV-2 XBB.1.5 challenge, co-housing, virological and tissue analysis. Naive hamsters received two intranasal doses of PBS as controls (n = 6) or AdC68-4RBD(XBB.1.5)-N (n = 6). Another group of naive hamsters (index) were infected to be virus donors. Created in BioRender. Xin, S. (2026) https://BioRender.com/ymdu7fb. **(B)** Viral loads in nasal turbinates and lungs measured by plaque assay and RT-qPCR. Data are means ± SEM; analyzed by one-way ANOVA with Sidak’s test (ns, p > 0.05; *p ≤ 0.05; **p ≤ 0.01; ***p ≤ 0.001; ****p ≤ 0.0001). **(C)** Immunofluorescence (upper) and H&E staining (lower) of nasal and lung tissues. Infected cells were detected using anti-NP antibody (green) and counterstained with DAPI (blue). Scale bar, 50 μm.

To assess long-term protective efficacy, Syrian hamsters were intranasally immunized with two doses of AdC68-4RBD(XBB.1.5)-N (2 × 10^10^ vp) at a four-week interval. Four months after the prime immunization, six unvaccinated index hamsters were intranasally challenged with 1 × 10^5^ PFU of live SARS-CoV-2 XBB.1.5 to serve as virus donors (Fig 6A). Two days post-challenge, each infected index hamster was co-housed for 4 hours with two contact hamsters from either the PBS control group (n = 6) or the vaccinated group (n = 6). Index animals were euthanized immediately after co-housing, while contact animals were euthanized two days later for tissue analysis (Fig 6A).

Index hamsters displayed high viral titers in nasal turbinates (NTs) and lungs (9.22 × 10^5^ and 9.53 × 10^5^ PFU/mL, respectively), confirming productive infection (Fig 6B). Vaccinated contact animals showed significantly reduced NT viral titers and viral RNA levels compared to PBS controls. While lung viral titers were comparable between groups, vaccinated animals exhibited marked reductions in lung viral RNA (1.6-log) and subgenomic RNA (1.3-log) (Fig 6B).

Immunofluorescence and H&E staining revealed abundant viral antigen and tissue damage in NTs of index and PBS control animals, and in lungs of index animals. In contrast, vaccinated animals had no detectable viral antigen or histopathological changes (Fig 6C). These results demonstrate that intranasal AdC68-4RBD(XBB.1.5)-N provides robust and durable protection, lasting at least four months, against SARS-CoV-2 XBB.1.5 replication and transmission in vivo. However, residual virus detected in vaccinated contact hamsters indicates that protection against transmission was substantial but not complete, which might be related to a potential decrease in IgA levels, as inferred from their temporal decline in BALB/c mice (Fig 3D).

### Intranasal AdC68-4RBD(XBB.1.5)-N protects K18-hACE2 mice against SARS-CoV challenge

**Fig 7:**
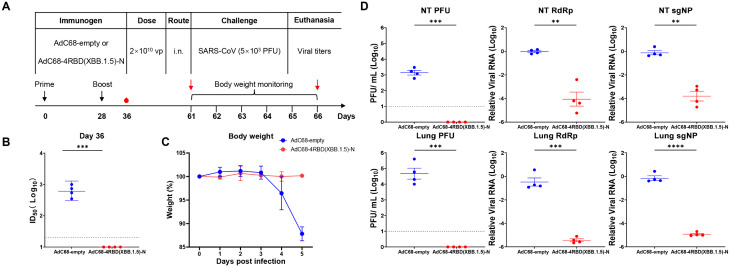
Intranasal AdC68-4RBD(XBB.1.5)-N protects against SARS-CoV infection in K18-hACE2 mice. **(A)** Experimental timeline of vaccination, serum collection, SARS-CoV challenge, body-weight monitoring for 5 days, and euthanasia for tissue analysis (n = 4 per group). **(B)** Serum neutralizing titers (ID_50_) at day 36 post-prime against the SARS-CoV pseudovirus. Data are geometric means ± 95% CI; analyzed by unpaired Welch’s t-test (two-tailed) (***p ≤ 0.001). **(C)** Body weights were recorded daily for up to 5 days post-challenge, normalized to day 0. **(D)** Viral loads in nasal turbinates and lungs measured by plaque assay and RT-qPCR. Data are means ± SEM; analyzed by unpaired Welch’s t-test (two-tailed) (**p ≤ 0.01; ***p ≤ 0.001; ****p ≤ 0.0001).

To further evaluate protective efficacy against more distant SARS-CoV infection, K18-hACE2 transgenic mice were intranasally immunized with two doses of AdC68-4RBD(XBB.1.5)-N (2 × 10^10^ vp) at a four-week interval and subsequently challenged with SARS-CoV (Fig 7A). Day 36 sera from vaccinated mice showed potent SARS-CoV pseudovirus neutralization, whereas sera from AdC68-empty–immunized animals were below the limit of detection (Fig 7B). At day 61, animals were intranasally challenged with 5 × 10^3^ PFU of authentic SARS-CoV. Body weight was monitored daily through day 5 post-infection (Fig 7A). Vaccinated mice maintained stable body weight throughout the observation period, whereas control mice exhibited progressive weight loss (Fig 7C). Viral loads in respiratory tissues were significantly reduced in vaccinated animals compared to controls, as measured by plaque assay and RT-qPCR for both genomic and subgenomic RNA (Fig 7D). These results demonstrate that intranasal AdC68-4RBD(XBB.1.5)-N provides strong protection, effectively preventing clinical disease and reducing SARS-CoV replication in the respiratory tract. Combined with the durable protection against SARS-CoV-2 XBB.1.5 observed in hamsters, these findings validate the heterologous tandem-RBD strategy for achieving broad protection across pathogenic coronaviruses.

## Discussion

Current intramuscular COVID-19 vaccines reduce severe disease but fail to prevent infection, allowing viral persistence, immune escape, and transmission. Here, we developed an intranasal vaccine using the chimpanzee adenovirus vector AdC68, which has low pre-existing seroprevalence in humans and a favorable safety record [20–23], and demonstrated durable, protective immunity in mice and hamsters. To broaden coverage against major pathogenic coronaviruses, we engineered heterologous tandem-RBD constructs by linking RBDs from SARS-CoV-2, SARS-CoV, and MERS-CoV in a head-to-tail configuration, fused to the SARS-CoV-2 nucleocapsid (N) protein to enhance T cell responses. These constructs preserved structural integrity and key epitopes, as confirmed by binding to human receptors ACE2 and DPP4 and to multiple conformation-sensitive RBD-specific monoclonal antibodies. In mice, comparative immunogenicity studies identified AdC68-4RBD(XBB.1.5)-N as the most potent candidate, inducing durable mucosal and systemic humoral immunity as well as tissue-localized B and T cell responses. Bone marrow antibody-secreting cells (ASCs) collected 22 weeks post-prime produced broad and strain-specific neutralizing antibodies, offering mechanistic insight into the breadth and durability of the antibody response. In hamsters, intranasal AdC68-4RBD(XBB.1.5)-N conferred strong protection against respiratory transmission of live SARS-CoV-2 XBB.1.5 even four months after vaccination. Robust protection was further demonstrated in K18-hACE2 transgenic mice against SARS-CoV challenge, confirming the broad efficacy. These results demonstrate that intranasal AdC68-4RBD(XBB.1.5)-N can block viral replication and transmission in vivo, highlighting its potential as a next-generation mucosal vaccine with broad and durable protection.

A key advantage of the AdC68 platform is its low pre-existing immunity and proven safety in humans, in contrast to high-seroprevalence vectors such as AdHu5 [20–23]. Chimpanzee adenoviruses—including AdC68, ChAdOx1, ChAd63, ChAd3, and ChAd36—efficiently infect epithelial cells at respiratory and gastrointestinal surfaces and have been used to deliver antigens from diverse viral pathogens [2,3,11–13,35–43]. Several have advanced to clinical use, including a nasal ChAd36-based SARS-CoV-2 vaccine authorized in India [44]. Our findings build on this evidence, showing that an intranasal AdC68 vaccine co-expressing tandem RBDs and N antigen provides durable protection against infection and transmission, supporting its potential for broad, long-lasting respiratory virus immunity.

An important feature of intranasal AdC68-4RBD(XBB.1.5)-N is its exceptional durability. In mice, systemic and mucosal responses persisted for at least 40 weeks, and in hamsters, protection was maintained four months after immunization. In contrast, many candidate vaccines elicit short-lived immunity, requiring multiple doses to maintain detectable neutralizing titers or protection [45–48]. This durability is particularly relevant for respiratory pathogens that spread via mucosal surfaces. Intranasal delivery also offers practical advantages over repeated injections, potentially improving vaccine uptake. The sustained immunity likely reflects both the broad cell tropism of AdC68 and the stability and epitope diversity of the tandem-RBD design, which outperformed monomeric, trimeric, or mixed RBDs. Beyond robust IgG and IgA responses, we identified broad and strain-specific mAbs from bone marrow ASCs 22 weeks post-prime, with a substantial fraction cross-neutralizing SARS-CoV-2 and SARS-CoV. Using our MoSMAR-chip platform, we directly isolated antigen-specific ASCs and characterized long-lived plasma cells in bone marrow, overcoming limitations of conventional memory B cell assays that rely on membrane-bound BCRs absent in ASCs [31].

The heterologous tandem-RBD design offers significant potential for cross-sarbecovirus and cross-merbecovirus protection. Our neutralization data demonstrate that AdC68-4RBD(XBB.1.5)-N elicits antibody responses targeting all three pathogenic coronaviruses, though with varying potencies reflecting the immunodominance hierarchy among the tandem RBD components. The SARS-CoV and MERS-CoV specific neutralization titers were higher than SARS-CoV-2.

Importantly, vaccination in hamsters and the K18-hACE2 mice conferred protection against SARS-CoV-2 XBB.1.5 and SARS-CoV. This broad protective efficacy might be supported by the dominant IGHV14–3/IGKV14–111 antibody lineage, which exhibited broad cross-reactivity. For MERS-CoV, we observed durable neutralizing antibody responses and identified MERS-CoV–specific mAbs from bone marrow ASCs, suggesting that the vaccine induces MERS-CoV–directed immunity. However, as MERS-CoV utilizes DPP4 rather than ACE2, in vivo challenge studies require hDPP4-transgenic animal models that are not available for this study. Instead, the mucosal IgA levels observed in intranasally immunized animals were measured using the 4RBD antigen, which contains epitopes from all three coronaviruses. Therefore, the IgA pool is expected to include antibodies with cross-reactive and virus-specific activities, like the IgG pool. Whether the mucosal IgA levels are sufficient to block MERS-CoV at the respiratory surface remains to be investigated.

This study has limitations. Immune responses were evaluated primarily in homologous settings; heterologous prime-boost regimens remain unexplored. The structural basis for cross-neutralization by isolated mAbs warrants further investigation. The small panel of bone marrow–derived mAbs from a single immunized animal may limit generalizability. Finally, as only a recombinant AdC68 vector was assessed, findings may not translate to other viral vectors or mRNA platforms. Comparative studies across vaccine modalities and broader coronavirus panels will be important to guide future development.

In summary, AdC68-4RBD(XBB.1.5)-N combines durable mucosal and systemic immunity with strong protection against viral replication and transmission, as demonstrated by SARS-CoV-2 XBB.1.5 protection in hamsters and SARS-CoV protection in K18-hACE2 mice. Its heterologous tandem-RBD design preserves key neutralizing epitopes across pathogenic coronaviruses, offering a rational strategy to enhance both breadth and durability. Coupled with the practicality of intranasal delivery, these attributes position this vaccine as a promising candidate for next-generation mucosal immunization against diverse human and animal coronaviruses.

## Materials and methods

### Ethics statement

All animal experiments were performed in accordance with the Guide for the Care and Use of Laboratory Animals of the People’s Republic of China and approved by the Animal Ethics Committees of Tsinghua University and The University of Hong Kong. Mouse immunization and characterization were conducted in the animal facility of Tsinghua University. Animal protection assays including hamster and K18-hACE2 mouse challenges were conducted in the BSL-3 facility of The University of Hong Kong.

### Protein expression and purification

Coding sequences for coronavirus RBDs and their tandem/trimeric constructs were designed as summarized in S1 Table. All constructs contained an N-terminal tPA signal peptide and a C-terminal 8 × His tag, and were cloned into the pVRC8400 vector for expression in HEK293F cells. Five days post-transfection, supernatants were harvested and purified using Ni-NTA Sepharose (Qiagen, Cat. #30230). Recombinant human ACE2-Flag-Strep (residues S19–S740) and DPP4-Strep (residues S39–P766) were expressed in the same system and purified with Strep-Tactin Sepharose (IBA-lifesciences, Cat. #2-1201-025). Final purification was performed by gel filtration chromatography (Superdex 200 Increase 10/300 GL, Cytiva) in PBS, and protein concentrations were determined spectrophotometrically.

### Structural prediction and validation of recombinant 3RBD and 4RBD by AlphaFold3 and SPR

The structures of 3RBD and 4RBD recombinant proteins were predicted by AlphaFold3 and visualized in Chimera [49,50]. Binding to receptors ACE2 and DPP4, and to conformation-sensitive mAbs was measured by a Biacore 8K SPR system (Cytiva). Specifically, anti-His antibodies (Cytiva) were immobilized on a CM5 sensor chip by amine coupling in 10 mM sodium acetate buffer (pH 4.0), yielding ~8500 response units (RU). His-tagged recombinant proteins were then captured on the chip surface. For affinity measurements, recombinant receptors and mAbs were injected at graded concentrations over CM5 chips functionalized with nine recombinant proteins, followed by a dissociation phase. Association (k_a_) and dissociation (k_d_) rates were determined, and equilibrium dissociation constants (K_D_) were calculated as k_d_/k_a_.

### Construction, amplification, and purification of recombinant AdC68-based vaccines

The rare-serotype chimpanzee adenovirus 68 (AdC68) vector was generated by deleting the E1/E3 regions and partially replacing E4 with AdHu5 sequences [51]. Vaccine constructs encoded the protein configurations shown in S1 Table, with 4RBD(XBB.1.5)-N additionally incorporating the SARS-CoV-2 N protein (residues M1–A419, GenBank: QIG55861.1) via a P2A linker. Recombinant plasmids (pAdC68-3RBD, pAdC68-4RBD(BA.4/5), pAdC68-4RBD(XBB.1.5), and pAdC68-4RBD(XBB.1.5)-N) were linearized with PacI and transfected into HEK293A cells for virus rescue. An empty AdC68 vector (AdC68-empty) lacking an E1 insertion was used as a control. Replication-deficient viruses were amplified in HEK293A cells stably expressing E1, purified by CsCl gradient ultracentrifugation, and quantified by spectrophotometry.

### Confirmation of RBD and N protein expression by western blot and flow cytometry

HEK293T cells (ATCC) were seeded in 6-well plates and infected with AdC68-3RBD, AdC68-4RBD(BA.4/5), AdC68-4RBD(XBB.1.5), AdC68-4RBD(XBB.1.5)-N, or AdC68-empty at a dose of 10^10^ vp per well. After 24 h, cells were harvested and lysed in 200 μL lysis buffer (Beyotime, Cat. #P0013) supplemented with protease inhibitors (Beyotime, Cat. #P1005). Lysates were resolved by SDS-PAGE and subjected to western blotting with anti-SARS-CoV-2 RBD (1:4000, Sino Biological, Cat. #40592-T62) and anti-SARS-CoV-2 N (2 μg/mL, ABMAX, Cat. #05–0155) antibodies. HRP-conjugated anti-rabbit (1:5000, Promega, Cat. #W4011) and anti-mouse (1:5000, Promega, Cat. #W4021) secondary antibodies were used for detection. GAPDH (1:40,000, Proteintech, Cat. #10494–1-AP) served as a loading control, and AdC68-empty as a negative control.

For intracellular flow cytometry, infected cells were stained with SARS-CoV-2 RBD-specific mAbs (P2C-1F11, CB6, BD58–0459), a SARS-CoV RBD-specific mAb (S230), a SARS-CoV/SARS-CoV-2 RBD cross-reactive mAb (S309), a MERS-CoV RBD-specific mAb (VHH-227), and an anti-SARS-CoV-2 nucleocapsid antibody (AS95; Acro, Cat. #NUN-CH14), each at 10 μg/mL for 30 min at 4 °C. After washing, cells were incubated with PE-conjugated anti-human IgG (1:200, BioLegend, Cat. #410707) and analyzed on an LSRFortessa Cell Analyzer (BD Biosciences). VRC01, an HIV-1–specific antibody, was included as a negative control.

### Mouse immunization and sample collection

Fifty-four female BALB/c mice (6–8 weeks old) were randomly assigned into 12 groups (n = 4 or 5 per group). Recombinant monomeric RBD, trimeric RBD (foldon-fused), heterologous mixtures of monomeric and trimeric RBDs (from SARS-CoV-2, SARS-CoV, and MERS-CoV), and tandem RBD proteins (3RBD and 4RBD) were formulated with AddaVax (InvivoGen, Cat. #vac-adx-10) at a dose of 10 μg per mouse. PBS with AddaVax served as the control. Mice were immunized intramuscularly (i.m.) three times at 2-week intervals with 100 μL per injection.

One hundred female BALB/c mice (6–8 weeks old) were divided into 10 groups (n = 10 per group) and immunized with AdC68-based vaccines, including AdC68-3RBD, AdC68-4RBD(BA.4/5), AdC68-4RBD(XBB.1.5), AdC68-4RBD(XBB.1.5)-N, or AdC68-empty (2 × 10^10^ vp), administered via intranasal (i.n., 20 μL per dose) or intramuscular (i.m., 100 μL per dose) routes at 4-week intervals. In batch I, sera and saliva from five mice per group were collected longitudinally up to 40 weeks to evaluate antibody durability. In batch II, five animals per group were sacrificed for serum collection to assess antibody breadth. All serum samples were heat-inactivated at 56 °C for 30 min and stored at –80 °C prior to binding and neutralization assays.

Twenty additional female BALB/c mice (6–8 weeks old) were immunized intranasally or intramuscularly with AdC68-4RBD(XBB.1.5)-N or AdC68-empty (2 × 10^10^ vp) at 4-week intervals (n = 5 per group). At week 8 post-prime, lung, spleen, and bone marrow samples were harvested for antigen-specific B and T cell analyses. Lungs were minced and digested in HBSS (MACGENE) containing 2 mg/mL collagenase IV (Worthington, Cat. #LS004188) at 37 °C for 45 min, filtered through a 70 μm strainer, and subjected to 35% Percoll (Cytiva, Cat. #17-0891-01) density centrifugation. Spleen and bone marrow samples were homogenized in PBS with 2% FBS, filtered, and subjected to centrifugation. Cell pellets were treated with erythrocyte lysis buffer (MACGENE, Cat. #CC051.500), washed, and resuspended in RPMI 1640 medium for ELISPOT assays.

### Binding antibodies measured by ELISA

Antigen-specific antibody responses were assessed by ELISA. Ninety-six–well plates were pre-coated overnight at 4 °C with purified proteins (100 ng/well), including 3RBD, 4RBD(BA.4/5), 4RBD(XBB.1.5), and SARS-CoV-2 N protein (wild-type, residues M1–A419), all expressed in HEK293F cells. Plates were blocked at 37 °C for 1 h and then incubated either with serially diluted mouse sera (for IgG) at 37 °C for 1 h, or with 100-fold diluted sera and 10-fold diluted saliva samples (for IgA) at 37 °C for 1.5 h. After three washes with PBST (PBS containing 0.05% Tween-20), plates were incubated at 37 °C for 45 min with HRP-conjugated anti-mouse IgG (1:4000) or IgA (1:10,000; Abcam, Cat. #ab97235). Following three additional PBST washes, TMB substrate was added, and reactions were stopped with 1 M H_2_SO_4_. Absorbance was measured at 450 nm (OD_450_) and 655 nm (OD_655_) using an iMark Microplate Absorbance Reader (Bio-Rad, USA). Final values were calculated as OD_450_–OD_655_ (OD_450–655 nm_).

For monoclonal antibody binding assays, plates were pre-coated with recombinant RBDs (SARS-CoV-2 wild-type or XBB.1.5, SARS-CoV, and MERS-CoV; 100 ng/well). Antibodies were added at 2 μg/mL, followed by HRP-conjugated anti-human IgG (1:4000; Promega, Cat. #W403B). All subsequent steps were performed as described above.

### Pseudovirus neutralization assay

Pseudovirus production and neutralization assays were performed as previously described [17,52,53]. Briefly, pseudoviruses were generated by co-transfecting HEK293T cells with the HIV-1 backbone encoding firefly luciferase (pNL43R-E-luciferase) and pcDNA3.1 (Invitrogen, USA) expressing spike proteins from SARS-CoV-2 variants (D614G, Beta, Delta, BA.1, BA.4/5, XBB.1.5, XBB.1.16, EG.5.1), SARS-CoV, MERS-CoV and its variants (L411F, T424I, S459T, A482V, L506F, D509G, V530L, V534A, E536K, D537E), GD, and WIV16. Supernatants were collected after removal of cell debris and stored at –80 °C until use.

For neutralization assays, serial 3-fold dilutions of mouse sera or monoclonal antibodies were incubated with pseudoviruses at 37 °C in 5% CO_2_ for 1 h in 96-well plates. Huh7 cells (2 × 10^4^/well) were then added and cultured at 37 °C in 5% CO_^2^_ for 48 h. Luciferase activity was measured using the Bright-Lite Luciferase Assay System (Vazyme, China). The 50% inhibitory dilution (ID_50_) or concentration (IC_50_) was determined using Prism v8.3.0 (GraphPad, USA).

### B and T cell responses measured by ELISPOT

B cell responses were assessed via ELISPOT assay. ELISpot plates (MabTech, Cat. #3654-TP-10) were activated with 35% ethanol, washed with PBS, and then pre-coated overnight at 4 °C with purified 4RBD(XBB.1.5) protein (300 ng/well). Plates were blocked with RPMI 1640 medium at 37 °C for 4 h. Single-cell suspensions from the lung, spleen, and bone marrow tissues of immunized mice were added to the plates and incubated at 37 °C for 24 h. After five washes, ALP-conjugated anti-mouse IgG antibody (1:1,000; MabTech, Cat. #3310-4-1000) and HRP-conjugated anti-mouse IgA antibody (1:2,000) were added to each well and incubated for 1 h at room temperature. Following five washes, spots were developed using BCIP/NBT-plus substrate solution (for IgG) and KPL TrueBlue peroxidase substrate (for IgA), respectively. Spot enumeration was performed with an automated ELISPOT reader (AID, USA). Results are presented as antibody-secreting cells (ASCs) per 1,000,000 cells.

T cell responses were evaluated using an IFN-γ pre-coated ELISPOT kit (MabTech, Cat. #3321–4APT-10) according to the manufacturer’s protocol. Single-cell suspensions from the lung, spleen, and bone marrow tissues were stimulated with peptide pools (Genscript, 2 μg/mL of each peptide) covering SARS-CoV, MERS-CoV, SARS-CoV-2 RBD (WT and XBB.1.5 variant), or SARS-CoV-2 N protein. Phorbol myristate acetate/ionomycin and RPMI 1640 medium served as positive and negative controls, respectively. After incubation for 24 h at 37 °C, plates were washed five times, followed by sequential incubations for 2 h (biotinylated anti-mouse IFN-γ antibody) and 1 h (streptavidin-ALP) at room temperature. Following five washes, spots were developed using BCIP/NBT-plus substrate and quantified with an automated ELISPOT reader (AID, USA). Results are presented as spot-forming cells (SFCs) per 1,000,000 cells.

### Isolation of antigen-specific antibody-secreting cells from bone marrow

RBD-specific antibody-secreting cells (ASCs) were isolated from the bone marrow of an intranasally immunized mouse (AdC68-4RBD(XBB.1.5)-N) 22 weeks post-prime using the MoSMAR-chip platform [31]. Polystyrene particles (Spherotech, USA) were coated with RBD proteins (0.5 mg/mL) for 1 h at 4 °C and washed three times with PBS. Bone marrow ASCs were enriched using the CD138^+^ Plasma Cell Isolation Kit (Miltenyi Biotec, Cat. #130-092-530) and incubated for 1 h at 37 °C with RBD–coated polystyrene particles, AF561-labeled anti-mouse IgG (Thermo Fisher, USA), and APC-labeled anti-mouse IgA (Southern Biotech, Cat. #1165-11) on the MoSMAR-chip platform. RBD-specific ASCs were identified by fluorescence microscopy (Olympus, Japan), isolated with a microneedle, and transferred into lysis buffer in microwells.

### Cloning and expression of monoclonal antibodies

As previously described [31,54], variable heavy (VH) and light (VL) chain genes of mouse-derived mAbs were amplified by nested PCR and assembled into linear immunoglobulin expression cassettes to generate full-length IgG1 constructs. Paired heavy- and light-chain cassettes were co-transfected into HEK293T cells for binding screening. In parallel, VH and VL sequences were cloned into antibody expression vectors containing human IgG1 constant regions. For large-scale production, paired plasmids were transiently transfected into HEK293F cells. Antibodies secreted into culture supernatants were purified using AmMag Protein A Magnetic Beads (GenScript, USA), and concentrations were measured with a NanoDrop2000 (Thermo Scientific). Reference monoclonal antibodies, including S2H97, S309, eCR3022.7, 80R, S230, m396, CB6, BD58–0459, and VRC01 [55–63], as well as P2C-1F11, P2B-2F6, W305-2A4, W328-6G6, MERS-27, VHH-T71 (PDB: 8IDI), VHH-T148 (PDB: 8IDO), VHH-31 (PDB: 8IEE), and VHH-227 (PDB: 8IDM) isolated by our group [64–66], were expressed and purified using the same procedure as above.

### Genetic analysis of mouse-derived mAbs

Germline gene usage, somatic hypermutation (SHM), framework regions (FRs), and complementarity-determining region 3 (CDR3) loop lengths were analyzed using the IMGT/V-QUEST tool (http://www.imgt.org/IMGT_vquest/vquest). Genetic clustering and neighbor-joining phylogenetic trees were generated with MEGA v11.0.13 and refined using iTOL (https://itol.embl.de/). Germline pairing patterns of heavy and light chains were visualized as chord diagrams with the R package circlize, where arc width reflected the frequency of identified antibody pairs.

### Detection of S1 shedding from cell surface-expressed SARS-CoV-2 or SARS-CoV S glycoprotein

HEK293T cells transiently expressing wild-type SARS-CoV-2 or SARS-CoV spike (S) proteins on the cell surface were generated by transfecting 10 μg of plasmid DNA per 10-cm culture dish. At 48 hours post-transfection, cells were harvested and incubated at 37 °C with neutralizing antibodies (nAbs) at a concentration of 20 μg/mL for designated time periods (15, 30, 45, 60, 90, and 120 minutes). Recombinant human ACE2-Flag-Strep protein and the S2-specific monoclonal antibody C13C9 were included as positive and negative controls, respectively [67]. Following the designated incubation periods, antibody-treated cells were immediately transferred to ice and washed extensively with ice-cold PBS containing 2% FBS. For detection of bound antibodies and proteins, cells were stained for 30 min at 4 °C with PE-conjugated anti-human IgG secondary antibody (1:200 dilution, BioLegend, Cat. #410707) for nAb detection or PE-conjugated anti-Flag tag antibody (1:200 dilution, BioLegend, Cat. #637310) for ACE2 binding, and AF488 anti-HA.11 epitope tag antibody (1:200 dilution, BioLegend, Cat. #901509) for S1 protein N-terminal HA tag detection. After washing with ice-cold PBS containing 2% FBS twice, stained cells were resuspended and analyzed using an LSRFortessa Cell Analyzer (BD Biosciences).

Spike protein stability and conformational integrity were assessed by monitoring the dynamics of S1 mean fluorescence intensity (MFI) through N-terminal HA epitope signals following treatment with ACE2 protein or monoclonal antibodies. At each specified time point, the MFI of HA-positive cells was normalized relative to the MFI of control cells (without treated by antibodies or ACE2 proteins) to determine relative S1 protein retention on the cell surface.

### Surface plasmon resonance (SPR) analysis

His-tagged recombinant proteins were captured on CM5 chips via anti-His antibodies. For epitope mapping, receptors (ACE2 or DPP4) and mAbs were sequentially injected to evaluate whether they bound overlapping or distinct epitopes. To test competition between ACE2 or DPP4 and mAbs, receptors were injected onto the RBD-captured CM5 chip until equilibrium was reached, followed by antibody injection. Competition efficiency was quantified as the difference in antibody binding response units (ΔRU) between receptor-saturated and receptor-free conditions. Data analysis and curve fitting were performed using Biacore Insight Evaluation Software (GE Healthcare, v5.0.18.22102).

### Golden Syrian hamster immunization and SARS-CoV-2 challenge

Ten-week-old male Golden Syrian hamsters were obtained from the Centre for Comparative Medicine Research (CCMR), University of Hong Kong. Animals were housed under Biosafety Level 2 (BSL-2) conditions with ad libitum feed and water. Viral challenge experiments were conducted in a BSL-3 facility following standard SOPs.

For evaluating AdC68-4RBD(XBB.1.5)-N, 6 male hamsters were immunized intranasally twice with 2 × 10^10^ vp at a 4-week interval, while 6 additional hamsters received PBS as controls. Four months post-prime, 6 unvaccinated index hamsters were intranasally inoculated with 10^5^ PFU of SARS-CoV-2 XBB.1.5 (EPI_ISL_17205250). At 2 dpi, each index hamster was co-housed for 4 h with either two PBS-treated hamsters or two vaccinated hamsters. Index hamsters were euthanized immediately after co-housing. Contact hamsters were housed individually in new cages and euthanized 2 days post-co-housing.

Nasal turbinate and lung tissues were collected for viral load quantification by RT-qPCR targeting genomic (RdRp/Hel) and subgenomic (NP) RNA as previously described [68,69]. Infectious virus titers were determined by plaque assay: serial 10-fold dilutions of tissue homogenates were inoculated onto Vero-E6-TMPRSS2 monolayers in 12-well plates. Cytopathic effects were monitored at 3 dpi, and plaque-forming units (PFU) were calculated as number of plaques × dilution factor and expressed as PFU/mL of tissue homogenate.

### Histopathology and immunofluorescence staining

Nasal turbinate and lung tissues were fixed in 4% formaldehyde, paraffin-embedded, and sectioned for hematoxylin and eosin (H&E) and immunofluorescence (IF) analyses. H&E-stained sections were scanned and analyzed using the Akoya Vectra Polaris Automated Quantitative Pathology Imaging System (Akoya Biosciences, Marlborough, MA, USA). For IF, SARS-CoV-2 nucleocapsid protein (NP) was detected using an in-house rabbit anti-SARS-CoV-2-N antibody (1:5000), followed by an Alexa Fluor 488-conjugated goat anti-rabbit IgG (1:1000, Life Technologies), as previously described [69]. Sections were imaged with a Carl Zeiss LSM 780 confocal microscope, and images were analyzed using ZEN 3.3 software (Blue edition).

### K18-hACE2 mouse immunization and SARS-CoV challenge

For vaccination, 20-week-old male K18-hACE2 transgenic mice (n = 4 per group) received two intranasal doses of either AdC68-empty or AdC68-4RBD(XBB.1.5)-N at a 4-week interval with 2 × 10^10^ vp. On day 36, serum was collected to measure neutralizing titers against SARS-CoV pseudovirus. On day 61, mice were challenged with 5 × 10^3^ PFU of SARS-CoV (HKU-39849, GenBank: AY278491.2). Infected mice were monitored for body weight changes over 5 days (as recorded in S2 Table) and were euthanized for endpoint analysis on day 5 post-infection. Nasal turbinate and lung tissues were collected for viral load quantification via plaque assay and RT-qPCR targeting genomic (RdRp/Hel) and subgenomic (NP) RNA, as described above. All animal experiments involving SARS-CoV were conducted in a BSL-3 facility following standard SOPs.

### Statistical analysis

All statistical analyses and graphing were performed using GraphPad Prism 8.3.0. Half-maximal effective dilutions (ED₅₀), half-maximal inhibitory dilutions (ID₅₀) of mouse sera, and half-maximal inhibitory concentrations (IC_50_) of monoclonal antibodies were calculated using a five-parameter dose-response curve. Data presentation and details are provided in the figure legends. p-values were determined using unpaired Welch’s t-test (two-tailed) (ns, p > 0.05; *p ≤ 0.05; **p ≤ 0.01; ***p ≤ 0.001; ****p ≤ 0.0001) or one-way ANOVA with Sidak’s multiple comparison test (ns, p > 0.05; *p ≤ 0.05; **p ≤ 0.01; ***p ≤ 0.001; ****p ≤ 0.0001).

## Supporting information

S1 Table Coronavirus RBD constructs used in this study. (DOCX)

S2 Table Body weight monitoring of K18-hACE2 mice. (DOCX)

S1 Fig Binding kinetics of receptors and antibodies to recombinant RBDs measured by SPR. His-tagged RBDs (individual, trimer-foldon, and tandem) were immobilized on CM5 chips via anti-His antibodies. Serial dilutions of receptors and mAbs were injected for real-time binding analysis by SPR. Sensorgrams show experimental curves (colored) and 1:1 model fits (black). Ligand-analyte pairs are labeled.
(TIF)

S2 Fig Serum neutralization curves of AdC68-3RBD and AdC68-4RBD immunized animals. Neutralization was tested against pseudoviruses bearing spike proteins of 12 β-coronaviruses, including SARS-CoV-2 variants (D614G, Beta, Delta, BA.1, BA.4/5, XBB.1.5, XBB.1.16, EG.5.1), SARS-CoV, MERS-CoV, Pangolin CoV GD, and Bat CoV WIV16. Data represent two technical replicates. Dashed lines indicate 50% neutralization.
(TIF)

S3 Fig Isolation and characterization of RBD-specific bone marrow ASCs using a microwell array chip. (A) Immunofluorescence image of bone marrow ASCs from an AdC68-4RBD(XBB.1.5)-N–immunized animal, captured by RBD-coated particles in a microwell array chip. Cells were visualized with a fluorescent secondary antibody. Insets in the top panel are magnified below. Scale bars, 20 μm and 10 μm as indicated. (B) Competitive binding of test antibodies and ACE2 or DPP4 to individual RBDs immobilized via anti-His antibodies, measured by SPR. Green curves show antibody-only binding; blue curves indicate the test antibody binding in the presence of the receptor ACE2 or DPP4; orange curves represent baseline binding. (C) Half-maximal inhibitory concentration (IC_50_) of MERS-CoV specific neutralizing antibodies against MERS-CoV variants. (D) The dynamics of S1 MFI were assessed via N-terminal HA signals of the spikes after incubation with ACE2 protein or monoclonal antibodies. At each specified time point, the MFI of HA-positive cells was normalized to that of non-incubated control cells.
(TIF)
