# Supplementary material for: Broad and durable protection against SARS-CoV-2 and SARS-CoV by an intranasal chimpanzee adenovirus vaccine expressing tandem RBDs and nucleocapsid
Source: PLoS Pathog. 2026 Jul 24;22(7):e1014436. doi: 10.1371/journal.ppat.1014436 (PMC13399337; doi:10.1371/journal.ppat.1014436)
Supplement: S1 Table — (DOCX) [file ppat.1014436.s001.docx]

**S1 Table. Coronavirus RBD constructs used in this study.**

| **Construct** | **Composition (residues)** | **Notes** |
| --- | --- | --- |
| SARS-CoV-2 RBD | R319–K537 (GenBank: MN908947.3) | Prototype |
| SARS-CoV RBD | N318–V510 (GenBank: NP_828851.1) | Prototype |
|  |  |  |
| MERS-CoV RBD | E367–N592 (GenBank: AHI48572.1) | Prototype |
|  |  |  |
| RBD foldons | SARS-CoV-2 (R319–K537), SARS-CoV (R306–Q523), MERS-CoV (E367–Y606) fused with two G_3_S linkers + T4 fibritin trimerization motif | Trimeric forms |
| 3RBD | SARS-CoV-2 (R319–K537), SARS-CoV (R306–Q523), MERS-CoV (E367–N602) | Tandem |
| 4RBD(BA.4/5) | SARS-CoV-2 (R319–K537), SARS-CoV (R306–Q523), MERS-CoV (E367–N602), SARS-CoV-2 BA.4/5 (R319–K537, GISAID: EPI_ISL_12559461) | Tandem |
| 4RBD(XBB.1.5) | SARS-CoV-2 (R319–K537), SARS-CoV (R306–Q523), MERS-CoV (E367–N602), SARS-CoV-2 XBB.1.5 (R319–K537, GISAID: EPI_ISL_16283160) | Tandem |
|  |  |  |
