# Supplementary material for: Broad and durable protection against SARS-CoV-2 and SARS-CoV by an intranasal chimpanzee adenovirus vaccine expressing tandem RBDs and nucleocapsid
Source: PLoS Pathog. 2026 Jul 24;22(7):e1014436. doi: 10.1371/journal.ppat.1014436 (PMC13399337; doi:10.1371/journal.ppat.1014436)
Supplement: S2 Table — (DOCX) [file ppat.1014436.s002.docx]

**S2 Table. Body weight monitoring of K18-ACE2 mice.**

| **Body weight (g)** | **AdC68-empty** | | | | **AdC68-4RBD(XBB.1.5)-N** | | | |
| --- | --- | --- | --- | --- | --- | --- | --- | --- |
|  |  |  |  |  |  |  |  |  |
| 0 dpi | 32.25 | 33.33 | 30.31 | 24.01 | 23.5 | 30.87 | 33.15 | 27.07 |
|  |  |  |  |  |  |  |  |  |
| 1 dpi | 33.5 | 33.37 | 30.14 | 24.13 | 23.61 | 31.12 | 32.87 | 26.81 |
|  |  |  |  |  |  |  |  |  |
| 2 dpi | 33.66 | 33.63 | 29.97 | 24.15 | 24.72 | 30.78 | 32.94 | 26.64 |
|  |  |  |  |  |  |  |  |  |
| 3 dpi | 33.58 | 34.03 | 29.76 | 23.75 | 23.56 | 31.17 | 33.22 | 26.96 |
|  |  |  |  |  |  |  |  |  |
| 4 dpi | 33.38 | 33.8 | 27.41 | 21.71 | 22.8 | 31.26 | 33.45 | 27.31 |
|  |  |  |  |  |  |  |  |  |
| 5 dpi | 28.34 | 30.63 | 25.75 | 20.8 | 23.53 | 31.11 | 33.14 | 27.02 |
|  |  |  |  |  |  |  |  |  |
